# Evaluating the Quality and Comparative Validity of Manual Food Logging and Artificial Intelligence-Enabled Food Image Recognition in Apps for Nutrition Care

**DOI:** 10.3390/nu16152573

**Published:** 2024-08-05

**Authors:** Xinyi Li, Annabelle Yin, Ha Young Choi, Virginia Chan, Margaret Allman-Farinelli, Juliana Chen

**Affiliations:** 1 Discipline of Nutrition and Dietetics, Susan Wakil School of Nursing and Midwifery, Faculty of Medicine and Health, The University of Sydney, Camperdown, NSW 2006, Australia; 2 Charles Perkins Centre, The University of Sydney, Sydney, NSW 2006, Australia

**Keywords:** artificial intelligence, apps, dietary assessment, dietitian, dietetic practice, food, nutrition care process, mHealth, mobile phone, smartphone

## Abstract

For artificial intelligence (AI) to support nutrition care, high quality and accuracy of its features within smartphone applications (apps) are essential. This study evaluated popular apps’ features, quality, behaviour change potential, and comparative validity of dietary assessment via manual logging and AI. The top 200 free and paid nutrition-related apps from Australia’s Apple App and Google Play stores were screened (*n* = 800). Apps were assessed using MARS (quality) and ABACUS (behaviour change potential). Nutritional outputs from manual food logging and AI-enabled food-image recognition apps were compared with food records for Western, Asian, and Recommended diets. Among 18 apps, Noom scored highest on MARS (mean = 4.44) and ABACUS (21/21). From 16 manual food-logging apps, energy was overestimated for Western (mean: 1040 kJ) but underestimated for Asian (mean: −1520 kJ) diets. MyFitnessPal and Fastic had the highest accuracy (97% and 92%, respectively) out of seven AI-enabled food image recognition apps. Apps with more AI integration demonstrated better functionality, but automatic energy estimations from AI-enabled food image recognition were inaccurate. To enhance the integration of apps into nutrition care, collaborating with dietitians is essential for improving their credibility and comparative validity by expanding food databases. Moreover, training AI models are needed to improve AI-enabled food recognition, especially for mixed dishes and culturally diverse foods.

## 1. Introduction

In recent years, mobile health (mHealth) applications (apps) for diet and nutrition have gained popularity among the public, dietitians, and health professionals [1,2,3]. mHealth apps have become a powerful tool to complement and support nutrition care delivered by dietitians across the nutrition care process [4], such as in nutrition assessment, delivering nutrition and behavioural interventions, nutrition education, and food intake monitoring [4,5]. Reviews have shown that nutrition interventions delivered through mHealth apps, particularly alongside conventional care, can improve patients’ health outcomes, especially for chronic conditions and weight management [5,6,7,8], and can increase diet quality in participants [9].

In recent years, there has been accelerated interest in artificial intelligence (AI), particularly with the emergence of Chat Generative Pre-trained Transformer (ChatGPT) [10]. AI involves computer systems performing tasks that typically require human intelligence, such as learning, understanding, and applying knowledge [11,12]. In nutrition and dietetics research, as early as 2009 and 2013, studies reported exploring the use of AI in vision-based methods to automate dietary records and as a precursor to chatbots [13,14,15]. A recent review by Liang et al. [16] identified 177 ‘AI nutritionists’ from the literature, encapsulating intelligent software, wearable devices and sensors, web-based tools, and apps. These ‘AI nutritionists’ were found to have applications across different areas of nutrition care, including food recognition, dietary records, nutritional assessment, nutrient and recipe recommendations, and health monitoring (e.g., through practice management software and/or patient portals). Apps were the second most common form of AI nutritionist identified in this review, due to the increasing popularity of smartphones and the ease of obtaining food images for image recognition or active monitoring of chronic conditions [16]. AI integration in researcher-developed apps has been implemented to automatically recognise food and estimate nutrition content from images of foods, drinks and meals, to reduce the user burden of manual logging of foods [17,18], enhance meal detail accuracy, and reduce underreporting [19]. Chatbots are other popular AI examples that can be utilised to provide individuals with a personalised diet and nutrition education, based on their needs and preferences [20,21]. AI has the potential to enhance efficiency in nutrition care by analysing complex data, predicting disease outcomes, and offering personalised health and nutrition care, thereby allowing dietitians to then allocate more time for patient education and counselling [11].

Previous studies have extensively reviewed the quality of mHealth apps, focusing on overall app features and functionality alongside behaviour change potential, user engagement, and accuracy of dietary assessment, including energy and nutrient estimations [22,23,24]. These evaluations of apps, however, were undertaken prior to the accelerated growth in AI. As interest in AI-integrated apps continues to grow, there is a need to evaluate these features in commercially available smartphone apps for nutrition care. This ensures data accuracy and patient safety [11] and helps determine the appropriate contexts for AI use in nutrition care. Previous evaluations did not include analysis of AI features such as food identification and nutrient estimation from food image recognition.

Samad et al.’s paper in 2022 was one of the first to evaluate AI integration in food-tracking apps, including the ability of apps to identify foods and their volumes; however, only one app was identified as containing AI [25]. This study recommended that future AI-related design considerations include greater automation in meal logging, the use of comprehensive, enriched food composition databases, and more dietitian involvement.

With the AI landscape evolving rapidly over the past two years, further evaluation of AI features and integration in apps is warranted. Furthermore, a research gap is presented given that Samad et al.’s study did not assess the comparative validity of energy and nutrient outputs from the apps, the accuracy of AI-enabled food identification, nor determine their behaviour change potential for nutrition interventions [25]. Therefore, the aim of the current study was to evaluate the features, quality, and potential for behaviour change of popular commercially available nutrition-related apps to determine their suitability for use in the nutrition care process. Furthermore, this study aimed to determine the comparative validity of dietary assessment from apps with manual food logging and AI-enabled food image recognition.

This paper has been structured to describe the methodology undertaken for this app evaluation according to each element of the aim; the results are then presented as follows: the apps’ features; an assessment of the quality of the apps, including their behaviour change potential; the comparative validity of dietary assessment from manual logging apps and those with AI-enabled food image recognition; and the accuracy of AI-enabled food image recognition. These results are discussed in the context of previous literature and practice implications, and future directions have been described for dietitians, app developers, and regulatory bodies.

## 2. Materials and Methods

There were four phases to the selection and evaluation of apps for (1) metadata and feature extraction; (2) evaluation against the Mobile App Rating Scale (MARS) [26] and App Behaviour Change Scale (ABACUS) [27] tools; (3) analysis of the comparative validity of nutritional output data; (4A) AI-enabled food recognition; and (4B) comparative validity of automated nutrition output. These are described further in the sub-sections below.

### 2.1. Eligibility Criteria

The initial sample was obtained from the 200 top-ranked apps in both free and paid “Health and Fitness” category of the Apple App Store and Google Play Store in Australia. Duplicate apps between the two stores were removed. The specific eligibility criteria for each of the four phases is described in Table 1.

### 2.2. Data Extraction

#### 2.2.1. App Metadata and Features

For both free and paid apps, app metadata, as outlined by the respective Apple App Store or Google Play Store, were extracted using a pre-determined data extraction form. If the app was found in both stores, data from both stores were extracted. Data extracted included name, country of origin, rating, number of ratings, and number of downloads, ranking, description, and involvement of healthcare professionals in app development. Additional data such as the presence of in-app purchases/subscriptions and price, app features, images, and videos where previews are available, and evidence of the inclusion of AI were also noted. Specific purpose or target of an app (e.g., diet, disease) and the areas of the nutrition care process addressed were also extracted.

#### 2.2.2. App Quality Assessment Using the MARS and ABACUS Tools

Where apps were available on both the Apple and Google Play stores, the apps were only used on an Apple iPhone, otherwise, if it were a Google Play-specific app, it was used on an Android device. The apps were downloaded and used for three days by three researchers to assess app quality against the MARS tool [26], which examined the engagement, functionality, aesthetics, information quality, and overall subjective quality scale of the apps. The subjective quality scale and app-specific mean from the MARS tool were not included for analysis in this study to minimise the impact of personal biases on the scores and for greater standardisation. The ABACUS tool [27], a standardised tool made up of 21 items, was used to assess the potential to promote positive behavioural change covering the following four domains: knowledge and information, goals and planning, feedback and monitoring, and action.

#### 2.2.3. Comparative Validity of Nutritional Output from Manual Food-Logging Apps

Sample food records for three dietary patterns were created by dietitians: Western diet, Asian diet, and Recommended diet (based on the Australian Dietary Guideline recommendations) (see Supplementary Table S1). The three-day food records for all three dietary patterns were entered into the apps by three researchers in an alternating rotation (e.g., researcher 1 entered Western diet for Fastic (version no. 1.165.0), Asian diet for Hitmeal (version no. 1.34), Recommended diet for Noom (version no. 12.9.0), etc.). Nutritional data including energy, macronutrients (protein, total carbohydrate, total sugar, total fat, saturated fat, alcohol) and micronutrients (dietary fibre, iron, calcium, sodium) produced by the apps were exported into Excel (version no. 2404).

#### 2.2.4. AI-Enabled Food Image Recognition and Comparative Validity of Automatic Energy Outputs

Day 1 of Western and Asian diets (See Supplementary Table S2) were modified and used for AI-enabled food image recognition testing. Images of the food and drinks from these diets were taken by researchers with a smartphone camera. For the seven mixed dishes such as ‘Eggs on Toast with Butter’, ‘Spaghetti Bolognese’, ‘Hamburger’, ‘Beef and Vegetable Stir Fry’, ‘Beef Pho’, ‘Bibimbap’, or ‘Pearl Milk Tea’, images of the complete dish were taken. The food and drink photos from these selected diets were taken at a 45-degree angle from the flat surface beneath the meal, taken 30 cm away from the dish with controlled background environment and lighting. All images for single component and mixed dishes (*n* = 22, 16 food images, and 6 drink images) were uploaded to selected apps, which had AI-enabled food image recognition functions for food logging (See Supplementary Figure S1).

### 2.3. Data Analysis

#### 2.3.1. App Metadata and Features

Classification and features extracted from app metadata were counted and tabulated.

#### 2.3.2. App Quality Assessment Using the MARS and ABACUS Tools

To assess interrater reliability for app evaluation against the MARS and ABACUS tools, intraclass correlation coefficient (ICC) was calculated using IBM SPSS Statistics (version no. 29.0.1.0) for five randomly selected apps. MARS and ABACUS scores were tabulated and descriptive statistics including mean and standard deviation (SD) were calculated.

#### 2.3.3. Comparative Validity of Dietary Assessment from Manual Food-Logging Apps

All sample diets were entered into Foodworks.online (version no. 1; Xyris, QLD, Australia) [28]. Recipes not found in the Foodworks.online database were manually added from leading Australian supermarket chains (e.g., Coles) and popular online Asian recipe blogs (e.g., Poh’s Kitchen, Recipetineats). For food items that did not exist or when no appropriate substitute was available in the selected databases (AusBrands 2019 and AusFoods) in Foodworks.online, energy and nutrient data from the United States Department of Agriculture food composition database were collated and manually entered as a new food within Foodworks.online. The average from the three days of energy and nutrient output for the diet plans from Foodworks.online (hereafter referred to as three-day food records (3D-FRs)) was used as a benchmark to compare nutritional output from apps. This was undertaken in accordance with previous studies [22].

Reported energy from apps was tabulated, and the difference in energy compared to 3D-FRs was calculated. The difference in mean, standard error of the mean, and 95% confidence intervals were also calculated in Excel. Macronutrients were reported as a percentage of energy intake. Micronutrients were reported as density (mg/MJ energy intake), in accordance with a previous study [29].

#### 2.3.4. AI-Enabled Food Image Recognition and Comparative Validity of Automatic Energy Outputs

For the apps with AI-enabled food image recognition, if an app could detect a mixed dish correctly, such as recognising ‘Bibimbap’ (a Korean mixed dish consisting of rice, vegetables, and usually a source of protein), then all the main food components within that dish were marked as correctly identified. The recipe this study used had six food components, which were brown rice, zucchini, bean sprouts, spinach, grated carrot, and gochujang paste. If the app could only detect a few main food components within the combined dish, such as ‘brown rice’ and ‘spinach’, then it was deemed to detect two out of the six food components within the dish. Each app’s detection for all food components (*n* = 39) was tested and extracted for accuracy of food image recognition. Accuracy was calculated as the number of food components correctly identified by the app divided by the total number of food components.

Each mixed dish recipe with exact weight and ingredients was entered into Foodworks.online as individual recipes, and nutritional data were recorded. Packaged, single food or drink items were entered as is or nutritionally similar options from the Foodworks.online database were used where possible. If there were no appropriate options, especially for some of the Asian-diet foods/drinks, energy and nutrient data were obtained from Australian supermarket websites (Coles and Woolworths), and for the pearl milk tea, Gongcha’s website. The reported energy from AI-enabled image recognition for each mixed and single dish and the energy for each food component of dishes were then compared to relevant nutritional data analysed from Foodworks.online to assess accuracy. The energy reported by the AI-enabled food image recognition feature of apps was tabulated, and the difference in energy compared to values from the corresponding foods and mixed dishes recorded in the food records was calculated. The mean of difference and standard error of the mean for single dishes were calculated in Excel.

## 3. Results

### 3.1. App Metadata and Features

#### 3.1.1. Sample Characteristics

**Figure 1:**
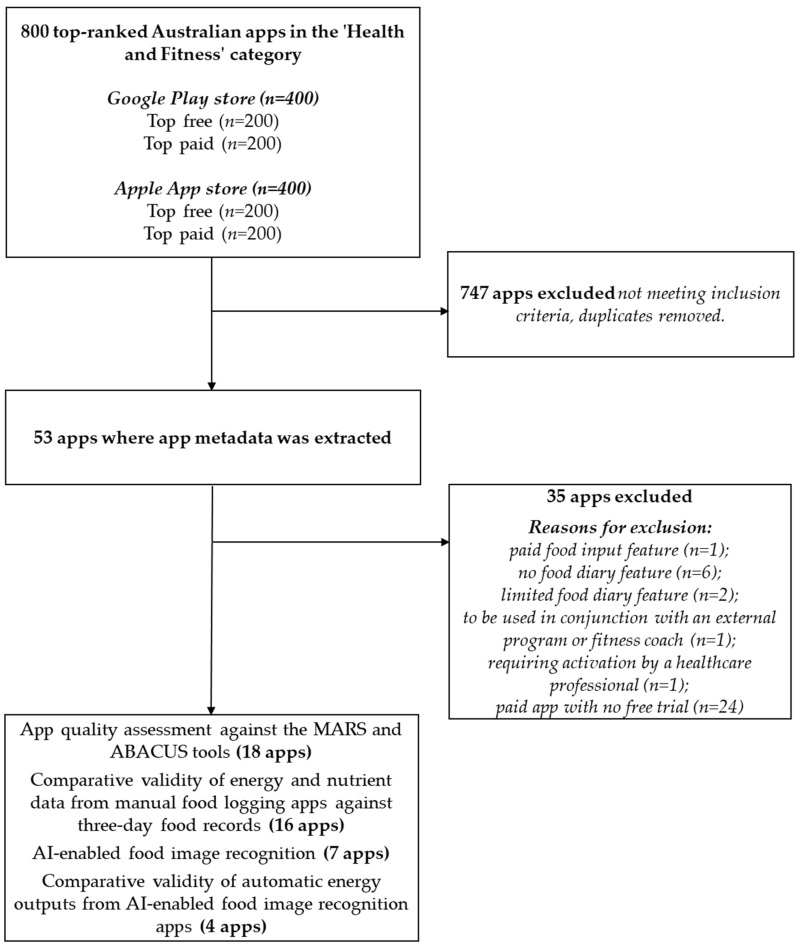
Flow chart of included and excluded apps for evaluation and analysis.

A total of 800 apps were screened from the Apple app store (200 free and 200 paid), and the Google Play store (200 free and 200 paid) against the app metadata eligibility criteria, resulting in 53 apps, where the app metadata were extracted (Figure 1). Eighteen apps included a food logging function; however, two apps (Balance (version no. 1.7.8) and WeightWatchers (version no. 10.60.0)) did not provide quantitative nutritional data. Nutritional data from the remaining 16 apps were extracted and evaluated for comparative validity against the 3D-FRs. Seven apps included AI-enabled food image recognition.

#### 3.1.2. App Metadata

Table 2 shows the metadata extracted from the 53 nutrition-related apps. Twenty-nine (55%) of the 53 apps were free to download, with nine of these requiring further payment or subscription to use. There were 24 (45%) apps requiring payment to download.

Food or nutrient tracking was the main purpose of 29 of the 53 apps (55%), with three apps tracking a specific nutrient or bioactive compounds (e.g., potassium, caffeine), five being primarily fitness apps, nine being nutrition-related educational resource or tools (e.g., Gluten Free Ingredient List (version no. 4.0)), and seven apps tracked other behaviours or outcomes such as fasting or weight. Twenty-three (43%) apps targeted a specific diet (e.g., Ketogenic diet (*n* = 2)); were associated with a program/product (*n* = 4) (e.g., Fitbit (version no. 4.13)), disease/disorder (*n* = 10) (e.g., diabetes), or lifestyle (*n* = 4) (e.g., intermittent fasting); or were specifically targeted towards women (*n* = 3) (e.g., menopausal women).

Thirty (57%) of the 53 apps indicated dietitian or healthcare professional involvement in either the development of the app, cited resources from scientific journals, or included blogposts (in-app or links to external webpages) written by dietitians. Limited information was found on what aspects dietitians were directly involved with in the design of the apps. Eleven (38%) out of the 29 apps that tracked food or nutrient intake included an integrated portal for healthcare professionals to manage patients.

Most apps had the potential to be integrated into nutrition assessment (*n* = 39), intervention (*n* = 38), and monitoring and evaluation (*n* = 45) steps in the nutrition care process. All the food tracking apps (*n* = 29) provide dietary information related to nutrition assessment and record basic anthropometric measures such as height, weight, age, and gender. Other notable assessment aspects included the app Nerva (version no. 29), which took a patient history of IBS, and many weight-loss-focused apps asked for a patient history regarding weight loss. Juniper (version no. 1.0.817) had Accredited Practising Dietitians (APDs) and general practitioners providing telehealth consults with the premium app users, which covers all aspects of the nutrition care process including nutrition diagnosis and intervention. Nutrition intervention strategies were mostly in the form of recipes or educational information relevant to an app’s purpose, e.g., diet, fitness, intermittent fasting, etc. Coaching and counselling features were observed in five apps, e.g., Fastic’s (version no. 1.165.0) AI chatbot and Noom (version no. 12.9.0). Forty-five apps could be used for nutrition monitoring and evaluation as most apps provided tracking features and the ability to review previous entries to identify trends and patterns.

#### 3.1.3. App Features

The features included for each of the categories (Dietary, Tracking, Insights, Technical, Education, Social, and AI) were defined in Table 3. These features were tallied for both free (*n* = 20), premium versions (*n* = 14), and paid apps (*n* = 19). The total number of features detected for each category is tabulated in Table 4 for free apps and Table 5 for premium versions and paid apps.

Premium versions of apps all provided more features than their free versions. For the apps with food diary features, the majority had multiple options for food input including text input and barcode scanning. Most apps had the ability to create custom foods and recipes, but many did not have the function to add timestamps. All apps except one (Blood Type Diet^®^ (version no. 2.6.8)) featured at least one tracking feature such as tracking weight, water, physical activity, fasting, or sleep. Most apps that were not educational tools (see ‘App Purpose’ in Table 2) included feedback on logged behaviours either as daily, weekly, or monthly breakdowns. Nineteen apps had recommendations for food or provided details on food quality using text or their own rating system.

There were 20 apps with a community feature but only 10 apps with the ability to add friends. Twenty-three apps included integration with external apps such as Apple Health or wearable devices. Thirty-seven (70%) of the 53 apps included additional information in the form of educational content, blog posts, or links to sources of information. Just under half of the apps (*n* = 24) included recipes, with the majority (*n* = 14) of these apps being paid apps or included as a feature of the premium version.

Regarding AI integration, four of the 32 free apps and 14 of the 35 paid or premium version apps included AI features. Only one app (Fastic premium version no. 1.165.0) included an integrated AI chatbot. Seven apps (13%) included AI-enabled food image recognition and seven apps clearly defined AI use for algorithmic calculations such as personalisation of energy expenditure or for meal plans. Notably, MacroFactor (version no. 2.6.8) used nutrition coaching algorithms to personalise calorie and macronutrient targets that continuously adjust according to user input of foods, exercise, weight, and calculated energy expenditure. It was unclear whether AI was used in algorithmic calculations for target weight projections or nutrient analysis in other apps. Other AI features identified were speech-to-text food input which indicated the use of multimodal large language models and “AI Describe” in MacroFactor (version no. 2.6.8), which implements natural language processing to log foods.

### 3.2. App Quality Assessment

#### 3.2.1. Inter-Rater Reliability

The Intraclass Correlation Coefficient (ICC) for MARS (Section A to D) and ABACUS were 0.72 and 0.66, respectively, indicating moderate interrater reliability across the three raters.

#### 3.2.2. MARS Tool

**Figure 2:**
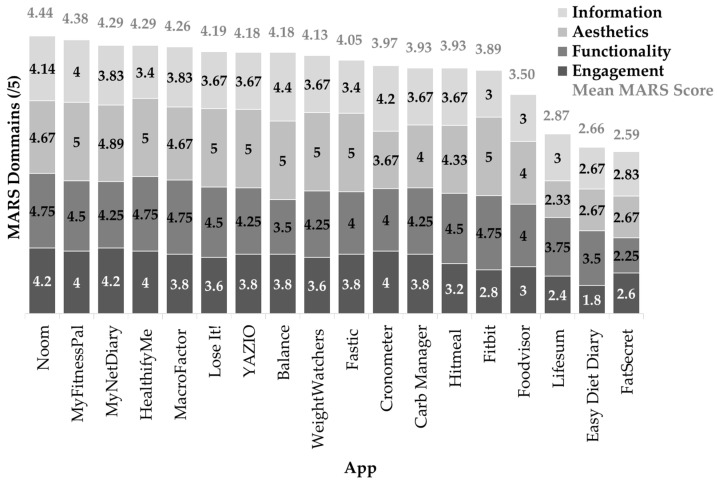
Scores for MARS section A–D (A: Engagement, B: Functionality, C: Aesthetics and D: Information) for nutrition-related apps (*n* = 18). Ranked from highest to lowest overall MARS score.

As indicated in Figure 2, Noom (version no. 12.9.0) received the top mean score of 4.44 in the MARS tool. The mean overall objective MARS score was 3.86 (SD 0.55). Fat Secret (version no. 9.32) scored the lowest in the MARS tool with a mean of 2.59 followed closely by Easy Diet Diary (version no. 6.0.28) (2.66) and Lifesum (version no. 18.3.0) (2.87). Overall, apps scored higher in the “Aesthetics” and “Functionality” domains, with an average score of 4.33 (SD 0.89) and 4.14 (SD 0.60) out of 5, respectively, and scored lower in the “Engagement” and “Information” sections, with an average score of 3.47 (SD 0.66) and 3.56 (SD 0.48) out of 5, respectively.

Mean scores for Question 1 (q. 1) were the lowest across all questions with a mean of 2.3 (SD 0.9) indicating limited entertainment features and gamification to increase engagement with the apps (see Supplementary Table S3). Collectively, most apps scored above a four out of five for the following questions: were appropriate for the target audience (q. 5), contained functional features (q. 6), and consistent and intuitive gestural design (q. 9). Ease of use and navigation functions of apps all scored three or above suggesting at least adequate usability except for FatSecret (version no. 9.32), which scored a one out of five for slow loading times between app pages and multiple incidences of crashing. Mean scores for all three questions in the “Aesthetics” section, which encompasses layout, graphics, and visual elements, were four or above. Sixteen of 18 apps (88%) scored four or above out of five for the clear and logical visual representation of data in the form of graphs, charts, or images. All apps included the ability to set goals (q. 14). For apps with educational elements, the information presented was mostly coherent, concise, and appeared correct (q. 15). Four apps (22%) did not include educational information within the app (q. 16). All apps excluding Cronometer (version no. 4.19.5) and Balance (version no. 1.7.8) scored one out of five for q.18 ‘credibility’. Ten apps (56%) have not been trialled or tested with conclusive evidence from published scientific literature with positive implications (q. 19).

#### 3.2.3. ABACUS Tool

**Figure 3:**
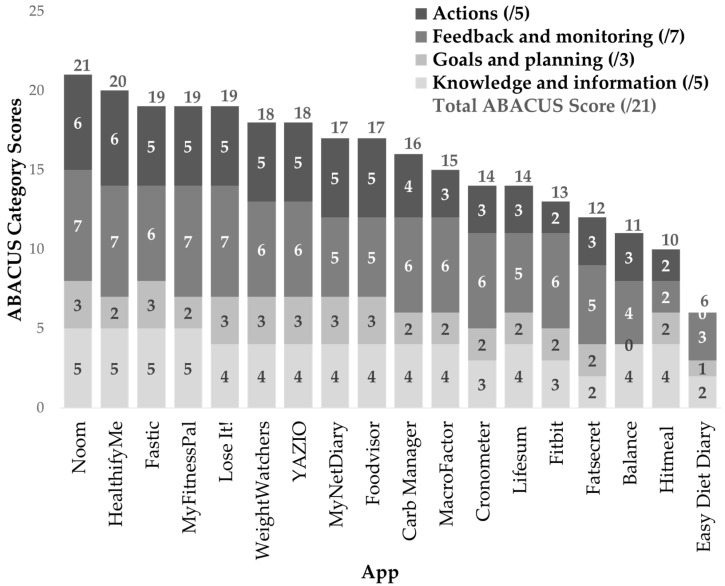
ABACUS category scores and overall scores for nutrition-related apps (*n* = 18).

Noom (version no. 12.9.0) received the top overall score of 21 out of 21 for the ABACUS tool (Figure 3). The mean overall ABACUS score was 15.5 (SD 3.9). Easy Diet Diary (version no. 6.0.28) scored the lowest with a score of six and a complete absence of measures from the “Actions” category. Apps scored higher in the “Knowledge and Information” and “Feedback and Monitoring” categories in the ABACUS tool, with apps collectively meeting 78% and 79% of the criteria, respectively, and lower in the “Goals and Feedback” and “Actions” categories, collectively meeting 69% and 62% of criteria, respectively.

All apps included the customisation and personalisation of features corresponding with measure 1.5 (e.g., notifications/reminders, food preferences, and unit system), recording of user baseline information (m. 1.3) such as height, weight, age, and BMI, and the ability to easily self-monitor behaviours (m. 3.2) through tracking food and exercise (see Supplementary Table S4). Over 15 apps (83%) of the 18 apps had a function to set goals (m. 2.2), e.g., for weight and/or exercise, and adjust these goals (m. 2.3), the provision of feedback on logged food diaries (m. 3.4), e.g., food and nutrient analysis, app-specific rating systems such as ‘Fastic Score’ or lesser-identified coaching/chatbot functions, and encouraged positive habit formation (m. 4.2) with various methods such as flexible calorie schedules, streaks, or badges and achievements. Additionally, 15 apps (83%) aided consumers in performing behaviours (m. 1.4). Notably, Noom (version no. 12.9.0) featured portion-size estimation guides, while Lifesum (version no. 18.3.0) provided information on vegetable and fruit serve sizes, and Fastic (version no. 1.165.0) provided extensive information on intermittent fasting and managing hunger. Ten apps exhibited the function to export data (q. 3.5). Only seven apps (39%) included information about the relationship between behaviour and possible consequences such as health, feelings, or cost (q 1.5). Seven apps (39%) identified consumers’ willingness for behaviour change (q. 2.1). The lowest scoring measure across the 18 apps was if the app provided solutions to restructure their physical or social environment (q. 4.5), with only five apps (27%) meeting this.

### 3.3. Comparative Validity of Dietary Assessment from Manual Food-Logging Apps

#### 3.3.1. Energy

**Figure 4:**
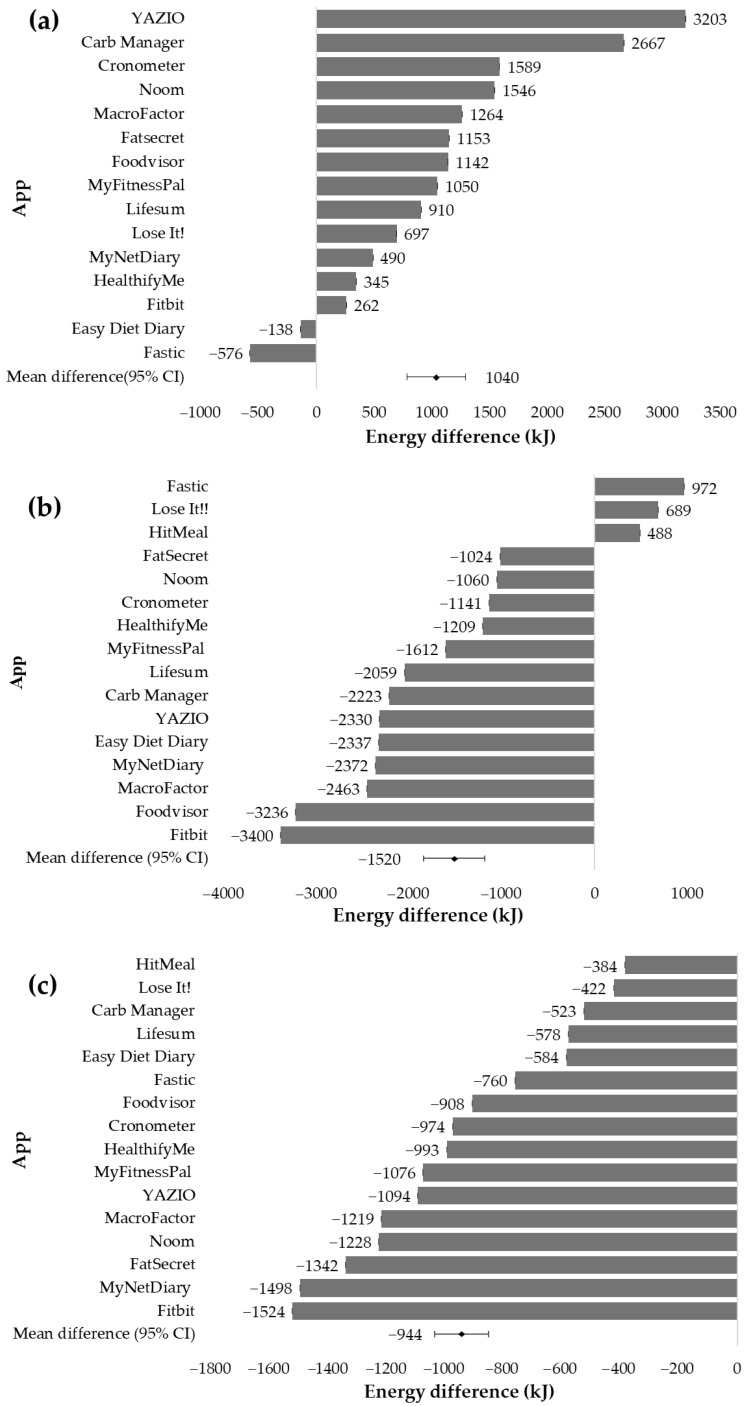
Energy difference based on three-day mean energy output from manual food-logging apps (*n* = 16) compared to three-day food records for the three diet types: (**a**) Western diet, (**b**) Asian diet, and (**c**) Recommended diet based on the Australian Dietary Guideline recommendations.

For the Western diet, 14 of the 16 manual food-logging apps overestimated energy intake, with a mean difference of 1040 kJ compared to the 3D-FRs (95% CI 544 to 1537), as depicted in Figure 4a. In contrast, for the Asian diet, 13 of the 16 apps underestimated energy, with a mean difference of −1520 kJ compared with 3D-FRs (95% CI −874 to −2165), as presented in Figure 4b. For the Recommended diet, all apps underestimated energy intake, and the mean difference was −944 kJ compared to 3D-FRs, (95% CI −762 to −1126) as shown in Figure 4c.

#### 3.3.2. Macronutrients

Macronutrients across the three diet types were provided by 15 of the 16 manual food-logging apps. Noom (version no. 12.9.0) only provided energy intake and, therefore, was not included in subsequent macronutrient and micronutrient data analysis. The Asian and Recommended diets did not include alcohol and, therefore, alcohol was omitted in their respective analysis.

For the percentage of energy from macronutrients in the Western diet, all 15 apps were comparable to the 3D-FRs (Table 6). Cronometer (version no. 4.19.5) and Fastic (version no. 1.165.0) had higher carbohydrate (%E) compared to 3D-FRs, 7% and 8% over the 3D-FRs, respectively. However, for the Asian diet, the mean of total fat intake (%E) across 14 apps was 6% over the 3D-FRs. For both Asian and Recommended diets, the mean carbohydrate intake (%E) across 14 apps was higher than the 3D-FRs (7% and 8% over 3D-FRs, respectively).

For the Asian diet, Fastic (version no. 1.165.0) reported an over 20% overestimation of carbohydrate (%E), whilst Lose It! (version no. 16.2.000) underestimated carbohydrate (%E) by 17% compared with the 3D-FRs. Lose It! (version no. 16.2.000) also underestimated total fat (%E) and saturated fat (%E) by about half of the 3D-FRs value (Table 6). For the Recommended diet, Fastic (version no. 1.165.0) overestimated saturated fat (%E) by a large amount, 14% compared to 7% of 3D-FRs (Table 6). Four of the sixteen manual food-logging apps reported alcohol intake, and three of the apps reported similar outputs to the 3D-FRs. MacroFactor (version no. 2.6.8) had the biggest discrepancy, underestimating alcohol (%E) by 3% compared to the 3D-FRs (Table 6).

#### 3.3.3. Micronutrients

Only six apps, namely, Cronometer (version no. 4.19.5), Easy Diet Diary (version no. 6.0.28), Fastic (version no. 1.165.0), Fitbit (version no. 4.13), Foodvisor (version no. 5.15.0-1), and MyFitnessPal (version no. 24.10.0) reported all four of the micronutrients: dietary fibre, calcium, iron, and sodium, in the free app version. Twelve manual food-logging apps provided nutritional data for at least one of the three micronutrients and were included in the micronutrient analysis (Table 7). All micronutrient data were reported as density (g or mg of micronutrients per Mega Joule of energy intake).

Reported dietary fibre density for the apps was overall similar to the 3D-FRs for all three diet types (Table 7). However, the calcium density reported for all three diets was poor in comparative validity. Iron density across three diet types was generally similar to 3D-FRs; however, Fitbit (version no. 4.13) reported a more than 20-fold higher iron density for the Western, Asian, and Recommended diets. For sodium density, Fastic (version no. 1.165.0) reported a more than 34-fold higher density ratio compared to 3D-FRs, for all three diet types. 

### 3.4. AI-Enabled Food Image Recognition

#### 3.4.1. Food Image Recognition and Identification Accuracy of Food Components and Mixed Dishes

In total, 22 images (comprised of 16 images of foods and six images of drinks) were uploaded into seven apps that had AI-enabled food image recognition features (Fastic (version no. 1.165.0), FatSecret (version no. 1.165.0), Foodvisor (version no. 5.15.0-1), HealthifyMe (version no. 11.1.0), HitMeal (version no. 1.34), Lose It! (version no. 16.2.000), and MyFitnessPal (version no. 24.10.0)). Within the 22 food or drink images, there were 39 total food components and seven mixed dishes. MyFitnessPal (version no. 24.10.0), Fastic (version no. 1.165.0), and HealthifyMe (version no. 11.1.0) had the highest accuracy of identifying food components using AI at 97% (*n* = 38/39), 92% (*n* = 36/39) and 90% (*n* = 35/39), respectively (Table 8). Lose It! (version no. 16.2.000) and FatSecret (version no. 1.165.0) had the lowest accuracy, each at 46% (*n* = 18/39).

#### 3.4.2. Comparative Validity of Automatic Energy Outputs from AI-Enabled Food Image Recognition Apps

Out of the seven apps with AI-enabled food image recognition, four apps (MyFitnessPal (version no. 24.10.0), Fastic (version no. 1.165.0), Foodvisor (version no. 5.15.0-1), and HealthifyMe (version no. 11.1.0)) automatically estimated energy values from the image-recognised food or drink. The AI-enabled food image recognition apps reported larger discrepancies in energy from single-component dishes compared to the food records (Table 9). MyFitnessPal (version no. 24.10.0) and Foodvisor (version no. 5.15.0-1) underestimated energy output (mean energy difference, −3% and −47%, respectively). HealthifyMe (version no. 11.1.0) and Fastic (version no. 1.165.0) had smaller differences compared to the food record of mean energy difference of 8% and 44%, respectively. Foodvisor (version no. 5.15.0-1) and Fastic (version no. 1.165.0) had the largest estimation discrepancy compared to the food record energy values (−47% and 44% mean energy difference, respectively). There were lower discrepancies for some single-component dishes including “Boiled Egg” and “Potato Chips”. “Boiled Egg” had overestimations of energy from 5% (Foodvisor (version no. 5.15.0-1)) up to 37% (HealthifyMe (version no. 11.1.0)). HealthifyMe (version no. 11.1.0) and Foodvisor (version no. 5.15.0-1) had a low underestimation discrepancy for “Potato Chips” from −12% to −16%, respectively, while other apps had large estimation discrepancies compared to the food record energy values.

For mixed dishes, there were notable discrepancies in the energy output produced by apps with AI-enabled food image recognition when compared to the food records (Table 10). For Western diet dishes, MyFitnessPal (version no. 24.10.0) showed a 35% underestimation for the “Eggs on toast with butter” image due to omitting butter, while Foodvisor (version no. 5.15.0-1) underestimated it by 73%. In contrast, for “Spaghetti Bolognese”, only MyFitnessPal (version no. 24.10.0) was able to identify three out of three food components in the image, while the other three apps did not identify the individual components of the dish.

For Asian diet mixed dishes, apps generally had greater difficulty identifying individual food components via image recognition. The individual components in “Beef and Vegetable Stir Fry” were identified by two apps (MyFitnessPal (version no. 24.10.0) and Foodvisor (version no. 5.15.0-1)), while MyFitnessPal (version no. 24.10.0) identified all four components, Foodvisor (version no. 5.15.0-1) identified three out of four components. For the dish “Bibimbap”, Foodvisor (version no. 5.15.0-1) and Fastic (version no. 1.165.0) were able to correctly identify some of the components (3/6 and 4/6, respectively). For other Asian diet dishes, such as “Beef Pho” and “Pearl Milk Tea”, apps failed to identify individual components, with significant underestimations of energy of up to 76%. 

## 4. Discussion

This evaluation revealed that most popular commercial smartphone apps for nutrition care available in Australia were food and nutrient trackers of acceptable to good quality. The apps showed variable behaviour change potential, and the overall provision of feedback, monitoring, and information was more common in apps, but the integration of goals, planning, and actions was lacking. There were notable discrepancies for both energy and macronutrients, especially for the Asian diet, compared to 3D-FRs, indicating poor comparative validity of manual food-logging apps. AI features were increasingly integrated, appearing in 7 out of 18 apps. These included image recognition of foods and drinks, voice-to-text meal logging, chatbots, and algorithmic calculations for energy expenditure personalisation. AI-enabled image recognition accurately identified food components, but energy estimation for mixed and cultural dishes was poor, highlighting the need for further model training and improvement.

Overall, apps for nutrition care had good functionality and aesthetics, with aesthetically pleasing layouts, graphics, and appeal. Limited features for engagement and varying information quality and credibility of information were observed. These findings align with other studies using the MARS tool to assess weight management and food or nutrition app quality [30,31,32]. Providing accountability and motivational support from dietitians is also necessary for more effective app engagement [4], particularly since loss of engagement with nutrition and diet apps is common [33,34,35].

It is a concern that the apps reviewed in this study showed deficits in credibility and a valid evidence base for educational content and advice. Most reviewed apps reported involvement from dietitians or nutritionists in app development, citing scientific journals and featuring in-app blog posts written by dietitians. However, the extent of dietitian involvement in app development and design was not readily provided. This aligns with other studies reporting insufficient scientific validation and healthcare professional involvement in nutrition apps [22,25,36,37,38]. App developers should be more transparent about the exact extent of dietitian involvement in app development and content creation. For dietitians, poor credibility is a major reason for not recommending mHealth apps [39]. Given the high patient interest in mHealth apps for nutrition care [1,40], increasing transparency of healthcare professional involvement in apps and dietitian training on app quality may increase confidence in integrating apps into dietetic practice and across the nutrition care process [4,41].

As is the case in the US and Canada, dietetic associations should review apps and advocate for app integration into dietetic practice to ensure acceptance across the profession [1,39]. Establishing such app libraries or repositories whereby developers can contribute their apps and have them evaluated by researchers or health professionals should be an initiative that is prioritised to ensure access to high-quality apps by public users. Unlike medical devices, the quality of mHealth apps has traditionally not been subject to regulatory oversight by governing bodies, such as the US Food and Drug Administration or Australia’s Therapeutic Goods Administration [22]. Over the past decade, government health departments (e.g., the UK National Health Service [42] and Australia’s Victorian Health (VicHealth) [43,44]) have adopted some responsibility for reviewing health apps to provide the public and healthcare professionals with guidance on the quality of different apps [45]. However, these efforts have not been sustained, presumably related to funding limitations. The National Institute for Health and Care Excellence has established an evidence standards framework for the commissioning or purchasing of digital health technologies, including AI, in the UK health and social care system [46]. The framework has clear standards around design, performance, value, and deployment, designed to support evaluators and decision makers, as well as companies in developing digital health technologies (e.g., apps, software, online tools, and programs for analysing data from medical devices or sensors) that benefit the population, individual service users, and/or the health and care system [46]. Therefore, a collaborative approach from dietitians, app developers, and regulatory bodies is needed for the development of practical mHealth apps by app developers and their endorsement by dietitians and regulatory bodies as a favourable tool in nutrition care.

Most apps showed potential for enabling behaviour change. Overall, apps scored higher in the “Knowledge and Information” and “Feedback and Monitoring” categories, and lower in the “Goals and Feedback” and “Actions” categories, which aligns with findings from other evaluations of nutrition and health apps using the ABACUS tool [43,47]. The lowest scoring ABACUS measure was whether the app provided solutions to restructure their physical or social environment, which was only addressed by 5 of the 18 apps (27%). Social and physical environments are important in supporting behaviour change [47] and can influence a person’s healthy food behaviour inclination [48]. While app developers should consider incorporating more behaviour change techniques into nutrition-related apps, it is important to recognise that some apps are primarily intended as electronic food records and not for instigating behavioural change. Therefore, dietitians should bridge this gap by providing professional guidance, evidence-based recommendations, and counselling that complements the behaviour change techniques offered in apps. For example, dietitians can focus on individual barriers, providing individualised and evidence-based strategies, feedback, and information, which were poorly covered by the apps for nutrition care assessed in this study [4].

This study identified discrepancies between the energy intake estimated from manual food-logging apps and 3D-FRs, with most apps overestimating energy for the Western diet. Other studies conducted in Western countries and with Western diets have also highlighted energy and nutrient discrepancies among popular commercial nutrition apps [22,24,49,50,51,52,53,54]. Most apps underestimated energy and fat intake and overestimated carbohydrate intake for the Asian diet. There is, however, a paucity of comparative validity evaluations examining Asian diets in apps [55,56]. For Japanese diets, MyFitnessPal significantly overestimated energy intake but underestimated total dietary fibre and sodium [55], whereas another study in the Philippines found that MyFitnessPal underestimated energy, carbohydrates, and fat and overestimated protein [56]. User input and crowdsourcing energy and nutrient data by commercial apps, such as MyFitnessPal, to their app databases, may further reduce the validity and reliability of nutritional output [52,56]. Moreover, most popular nutrition-related apps use the United States Department of Agriculture (USDA) food composition database, which typically contains food and drink from Western countries and offers limited Asian food and drink options [39,55]. This can make it difficult for users to choose a food item that exactly matches the foods they consumed and may lead to the selection of an alternate option such as a packaged frozen meal or takeaway meal. Furthermore, for many Asian foods, apps may have limited portion-size options, for example, only one standard takeaway box of Pad Thai noodles. At the time of data collection, it was identified that only Noom featured portion-size estimation guides, and Lifesum provided information on vegetable and fruit serve sizes. Given the findings, dietitians should verify commonly omitted foods and clarify portion sizes with patients when using popular apps for nutrition assessment [52].

Dietitians may also need to prescribe patients more accurate manual food-logging apps for the self-monitoring of specific nutrients and ensure patients are well trained in thorough food record entry. The clinical implications of using MyFitnessPal for weight management due to its underestimation of energy (445 kcal) have been previously raised [52]. Among the popular manual food-logging apps we evaluated, carbohydrates were overestimated by up to 20% of energy intake, particularly for the Asian diet. Such carbohydrate overestimation poses considerable risks (e.g., poor glycaemic control and hypoglycaemic episodes) for patients with diabetes who rely on carbohydrate counting to calculate fixed-dose insulin. In comparison, the accuracy of researcher-developed carbohydrate counting apps (e.g., GoCARB) is more comparable to dietitian-based calculations [57]. Dietitians should also be careful when prescribing these apps for patients who may self-monitor specific micronutrients for health. For example, compared to the 3D-FRs, the Fastic app reported sodium levels of more than 34-fold higher, which would create misinformation for a patient trying to manage their hypertension. The inconsistencies in sodium, calcium, and iron estimations have also been reported in another study of popular nutrition-related apps in the UK against 24 h weighed food records [58].

Dietitians have expressed that the digitalisation of food diaries is a desirable feature to support professional practice [39]. Despite being an improvement over traditional paper-based methods [4,33,59,60,61], the time commitment and patient burden from long-term use of food tracking apps remains a significant concern [39,62,63]. Integration of AI-enabled food image recognition offers a more streamlined and efficient method for automatic food logging which could enhance user experience by easing the burden of food logging and fostering sustained app engagement [3,19,64,65,66,67,68]. As of 2022, most commercial food-logging apps lacked automatic food recognition and volume estimation features, with Foodvisor being an exception [25]. At the time of this study, only two years on, the number of apps incorporating AI features has increased to seven, including AI-enabled food image recognition (four apps with AI-enabled volumetric and caloric estimation), and other AI features such as chatbots, voice-to-text meal logging, and algorithm-based personalised energy calculations. This is similar to another review where AI had applications in food recognition and dietary record (69%), nutrient and recipe recommendations (14%), health monitoring (14%), and other functionalities (3%) [16]. However, this review raised that the majority (68%) of existing nutrition-related software did not leverage AI integration in its truest sense. For example, typically, retrospective data are input into formulas, calculations, and existing rules to estimate nutritional values and provide recommendations, rather than harnessing AI through machine learning and deep learning to train predictive models for personalisation and active real-time monitoring [16].

To fully harness AI-enabled food image recognition apps in dietetic practice, particularly in dietary assessment, accurate detection of food and drink items and volume estimations are critical for valid energy and nutrient data. A high accuracy of 85% has been achieved for identifying pre-categorised food images when food image recognition models were trained on 50,000 food images using deep learning to recognise patterns [69]. In our current study, four of the seven apps with AI-enabled food image recognition achieved this identification threshold—namely, MyFitnessPal (97%), Fastic (91%), HealthifyMe (90%), and Foodvisor (87%). However, these four apps exhibited large discrepancies in their energy estimations when using AI-enabled food image recognition, particularly for mixed dishes. A factor contributing to these discrepancies included not detecting certain individual food components within the mixed dishes. Difficulties in identifying individual components within mixed dishes have been attributed to the variability of food appearance, type and shape, the containers they are placed in, or when foods or drinks may be similar in appearance but have varying nutritional profiles [70]. Furthermore, when multiple mixed dishes are present in a meal, for example in Asian, Mediterranean, or Middle Eastern cuisines, this can pose additional challenges in detecting all the foods present and limit usability for patients with culturally diverse diets. To overcome this, one study in Taiwan used a deep learning model to train a food recognition app to identify meals classified as single dishes, mixed dishes, and multiple dishes with high precision [71]. Moreover, the inability to detect ingredients added in cooking (e.g., butter, fat/oils, salt) or sauces and condiments is a recognised limitation of current food image recognition technology [72] and even manual food logging more broadly [52].

Incorrect estimation of portion sizes or volume can compound the inaccuracies associated with energy estimations automated food logging via AI-enabled food image recognition. To enhance the food image analysis process, images can be captured between a 45° and 60° angle, and the incorporation of a fiducial marker can assist with food identification and volume estimation [19]. This approach has been effective when participants were trained in how to capture images with a fiducial marker for a researcher-developed AI-enabled app for dietary assessment (FRANI) in Vietnam [73]. However, such studies using AI-assisted dietary assessment for Asian diets have emphasised that greater accuracy in nutrient outputs is achieved when users can also match foods and portion sizes that are not detected or are incorrectly identified through AI-enabled food image recognition [73,74]. However, for the public, training is not typically provided on how to capture images of food and drink, such as in research settings or during consultations with a dietitian. Commercial apps may therefore need to include an in-app tutorial on how to capture images for more accurate food image analysis.

### 4.1. Strengths and Limitations

A key strength of this study is that it extends previous evaluation studies of app quality by analysing app features, including AI integration. It is also the first to have specifically assessed the AI-enabled image recognition of food and drinks in commercially available apps for nutrition care for the accuracy of food components identified and the accuracy of energy estimation. Furthermore, this study examined the comparative validity of dietary assessment from apps across three culturally diverse diet plans, Western, Asian, and Recommended (based on the Australian Dietary Guideline recommendations) to allow representation of a range of cultural diet preferences. This was particularly undertaken as previous research has revealed that inadequate local food choices within apps and their food composition databases were major barriers to using nutrition apps for app users [52], non-users, dietitians, and health professionals [2,39].

The limitations of this study included funding constraints so only the free-to-use apps and features were included and evaluated. Hence, some advanced app features, especially AI features and additional nutrient data (e.g., micronutrients and macronutrients breakdown (like saturated fat, and added sugar), may have been missed from paid apps or premium upgrades where a free trial was not available. However, this is representative of what the majority of the public will utilise, as indicated by a previous study [24].

Moreover, the evaluation of AI-enabled image recognition accuracy was conducted in a controlled setting with good lighting, no background clutter, and at a consistent distance and angle. This may not be generalisable to how the public would capture images in free-living conditions where there is likely to be higher variance in image quality and so may impact recognition of the food components from the image, thereby possibly leading to greater inaccuracies [65]. While a fiducial marker was not utilised, which could have influenced the accuracy of the food image identification results, this has greater similarity with how apps might be used in a non-research setting.

The three researchers utilising the apps were young adults who are technology-savvy and trained in Nutrition and Dietetics. Young adults typically have higher levels of technological literacy and are more competent with digital technologies and smartphones [75]. Furthermore, the food logging and matching of food items in the apps were likely to be more accurate compared to public users because of the researchers’ nutrition knowledge and dietetics training.

### 4.2. Practice Implications and Future Directions

The implications on practice for dietitians, app developers, and regulatory bodies are summarised below in Box 1.

Box 1 **Practice implications and recommendations.**

For dietitians:

- Dietitians can provide professional guidance, evidence-based recommendations, and counselling that complements the behaviour change techniques offered in apps.
- Dietitians may need to prescribe patients more accurate manual food-logging apps for self-monitoring specific macronutrients and micronutrients and ensure patients are well trained in thorough food record entry.

For app developers:

- Consider incorporating more behaviour change techniques into nutrition-related apps.
- App developers should be more transparent about the exact extent of dietitian involvement in app development and content creation.
- Include in-app tutorials on how to capture images for more accurate food image analysis in AI-enabled food image recognition apps.

For regulatory bodies:

- mHealth apps are not governed by any specific regulatory body. Government health departments could play a role in the governance of these apps and undertake reviews to ensure the quality and safety of use by the public.
- Dietetic associations can undertake reviews of apps and advocate for app integration into dietetic practice to ensure acceptance across the profession.

Future studies should evaluate the accuracy of AI-enabled advice, including chatbots and personalised nutrition advice, in commercially available nutrition-related apps, against nutrition care provided by dietitians and guidelines for a healthy population and people with chronic health conditions or requiring support with weight management. Further analysis is necessary, particularly of AI features in paid and premium versions of apps to address the financial constraints encountered in this study.

App developers must intensify efforts to include dietitians on their development teams to expand food databases for diverse cultures and improve AI-enabled portion size detections. The comparative validity of the energy and nutrient data from apps across various multicultural diets should also be tested among a more diverse population (by age and culture) in free-living conditions to provide a more representative evaluation of app usability and accuracy.

User-generated images could be employed to train AI-enabled food image recognition models through deep learning, especially for mixed dishes and culturally diverse foods and drinks. Additionally, leveraging AI algorithms to process large datasets can aid in developing more accurate food image recognition for real-world applications. These considerations for future evaluations would provide a more in-depth analysis of apps for nutrition care to guide regulations and advocacy, and ultimately, increase their integration within the nutrition care process and dietetic practice.

In existing practice management software for nutrition care (e.g., Nutriadmin [76], Nutritio [77]), there is a trend of generative AI integration, such as ChatGPT for automated recipe generation. There is potential for generative AI to streamline processes that are time-consuming for dietitians, such as developing tailored meal plans based on patient preferences, existing diets, and health conditions and the personalisation of feedback and recommendations based on AI algorithms that combine information from different data sources, such as genomics, clinical, and health data. Future studies should evaluate the AI-enabled functions in software other than mobile applications.

## 5. Conclusions

Overall, the functionality and aesthetics of popular commercial smartphone apps for nutrition care in Australia have improved over the past decade, particularly in their AI features. Most apps have the potential to be integrated into the nutrition care process, particularly nutrition assessment, nutrition intervention, and monitoring and evaluation. Apps are still predominantly focused for use in dietary assessment as food and nutrient trackers and targeted for weight management. Issues related to the credibility of apps and ongoing inadequacies in dietitian involvement in app development remain a major concern. Substantial discrepancies and a lack of available nutritional data such as energy and macronutrients were observed, with higher levels of discrepancy for manual food logging and AI-enabled food image recognition from Asian dietary patterns. AI-enabled food image recognition was recognised as a feature beneficial to reduce patient burden. Future advancements in AI-enabled food image recognition require improvement in accuracy, especially for complex mixed and cultural dishes. This could be facilitated through training AI models with user-generated images and applying AI algorithms to process images. Furthermore, collaborations between dietitians, app developers, and regulatory bodies are essential for future app revisions and for the development of reliable, credible, and fit-for-purpose mHealth apps for professional use.

## Figures and Tables

**Table 1:** Eligibility criteria for the selection of apps.

Phase	Inclusion	Exclusion
(1) App metadata and features	(1) Any apps related to nutrition, e.g., caloric, macronutrients or micronutrients intake, weight management, or that could be incorporated into the nutrition care process (2) Were available in English.	(1) Researchers were unable to download the app. (2) The app was in a language other than English.
(2) App quality assessment using the MARS and ABACUS tools	(1) Have a food diary feature. (2) Free to download and use or free to download and have a free trial of the premium paid features.	(1) Apps where the food diary feature was a paid feature (due to funding constraints). (2) Apps without a food diary feature or there was an inadequate food diary feature (e.g., only provides set choices from an integrated recipe database or a description-only food diary, e.g., only provides the option to input size (‘small’, ‘medium’, and ‘large’), type (‘ketogenic’, ‘low carb’, ‘balanced, ‘high carb’, and other), or a textbox to ‘describe your meal’, or where the logged diary could not be accessed. (3) Apps intended to be used in conjunction with an external program or fitness coach. (4) Apps requiring activation by a healthcare professional. (5) Paid apps with no free trial.
(3) Comparative validity of dietary assessment from manual food-logging apps	The inclusion criteria as above in Phase 2 as well as: (1) Apps that included quantitative nutritional outputs from energy, macronutrients, and/or micronutrients.	The exclusion criteria as above in Phase 2, as well as: (1) Apps without quantitative nutritional outputs from energy, macronutrients, and/or micronutrients.
(4A) AI-enabled food image recognition	(1) Have an image-based food image recognition feature integrated as part of the food diary function (e.g., scanning a meal or dish, and the app automatically identifies the food or drink), which was accessible for free or under a free trial.	(1) Apps that had a food diary function but did not have image-based food image recognition features integrated. (2) Apps where the image-based food image recognition feature was a paid feature only (with no free trial).
(4B) Comparative validity of automatic energy outputs from AI-enabled food image recognition apps	(1) Apps included in the analysis of energy estimations from AI-enabled food image recognition against the food record reference were required to automatically estimate energy intake directly from image recognition of foods, drinks, or dishes and their portion sizes or volumes. (2) This feature needed to be accessible for free or under a free trial.	(1) Apps with AI-enabled food image recognition for automatic energy output as a paid feature (with no free trial).

**Table 2:** Metadata of the nutrition-related apps evaluated (*n* = 53).

Metadata	Item	Count (*n*) ^1^
Platform	Apple App Store Only Google Play Store only Both	11 0 42
Price	Free to download and use Free to download, requires payment to use Paid	20 9 24
Healthcare Professional	Dietitian/healthcare professional involvement ^2^ Integration with healthcare professional portal ^3^	30 11
App Purpose	Food or nutrient tracker Specific nutrient tracker Fitness app (primary) Educational resource or tool Tracker, other (e.g., fasting, weight)	29 3 5 9 7
Specific Target Area	Diet Disease/disorder ^4^ Program/product Lifestyle Women	2 10 4 4 3
Application to Nutrition Care Process Steps	Nutrition Assessment Nutrition Diagnosis Nutrition Intervention Monitoring and Evaluation	39 1 38 45

^1^ Note that some apps appear under multiple items. Counts do not reflect total unique apps. ^2^ Specifically, involvement from dietitians or nutritionists in either the development of the app, authors of blog posts or links to scientific evidence from credible sources (e.g., scientific journals, government websites). ^3^ Ability to manage patients in a portal specifically for healthcare professionals, direct contact, or exportation of data to a healthcare professional. ^4^ e.g., irritable bowel syndrome, coeliac disease, or diabetes.

**Table 3:** Features identified from app metadata.

Category	Features
Dietary	Individual food input Barcode food input Macronutrient breakdown Logging timestamps Custom food Create recipe or meal Meal plans Food diary (non-customisable or other) ^1^
Tracking	Water tracking (separate) Weight tracking Fasting tracker Physical activity or steps tracking Tracker (other) ^2^
Insights	Goal setting (weight or calorie) Goal setting (other) Daily/Weekly breakdown Food/nutrient analysis Food swap and/or recommendations Weight progress/trends Trends (other) ^3^
Technical	Integration with external apps/devices Export data from app Targeted for a specific diet, disease/disorder, program/product, lifestyle or women
Education	Additional information and/or articles Recipes
Social	Community forum and support Friend and/or buddy system
Artificial intelligence	AI chatbot Food recognition Algorithmic calculations Other

^1^ Included food diaries with a set recipe database, description-based meal logging, or categorical food diary (e.g., logging food groups). ^2^ Included trackers for sleep, vitamins, medicine, etc. ^3^ Other trend charts/graphs included water, physical activity, steps, fasting, sleep, etc.

**Table 4:** Number of features within free-to-use nutrition-related apps (*n* = 32).

App (Version No.)	Dietary	Tracking	Insights	Technical	Education	Social	Artificial Intelligence
Balance (1.7.8)	1	2	2	0	1	1	0
BMI Calculator (1.8.9)	N/A	N/A	N/A	0	0	N/A	0
BodyFast (3.35.3)	N/A	3	4	1	2	0	0
BodyMonitor (2.9.12)	N/A	N/A	2	2	0	N/A	0
Carb Manager (7.5.5)	6	3	5	2	1	2	0
Cronometer (4.19.5)	5	3	5	1	0	2	0
Easy Diet Diary (6.0.28)	5	3	4	2	0	0	0
FastEasy (1.39.2)	N/A	4	3	0	2	0	0
Fastic (1.165.0)	6	4	2	2	1	1	1
FatSecret (9.32)	5	3	5	2	1	1	1
Find Me Gluten Free (3.6.36)	N/A	N/A	N/A	0	1	N/A	0
Fitbit (4.13)	4	4	4	3	0	2	0
FoodSwitch (5)	N/A	N/A	3	0	0	N/A	0
Foodvisor (5.15.0-1)	5	3	3	1	1	0	0
HealthifyMe (11.1.0)	3	4	4	2	1	0	1
HitMeal (1.34)	3	3	4	1	0	0	0
Juniper (1.0.817)	0	2	2	2	2	0	0
Kahunas (2.1.0)	2	2	1	2	0	0	0
Keto Diet App (2.102)	6	4	5	2	2	1	0
Kic (3.3.8053)	2	2	0	0	2	1	0
Lifesum (18.3.0)	5	4	5	1	0	0	0
Lose It! (16.2.000)	5	2	3	1	1	2	0
MyFitnessPal (24.10.0)	5	2	6	1	2	2	0
MyNetDiary (9.11)	5	3	5	1	2	2	0
Nerva (29)	N/A	1	1	1	1	0	0
Omo (2.64.1)	3	5	3	2	2	1	0
Reverse Health (2.2.1)	3	4	3	0	2	1	0
Vitable (2.0.3)	N/A	1	N/A	0	1	N/A	0
WeightWatchers (10.60.0)	3	4	6	2	2	1	1
YAZIO (10.6.1)	5	3	5	2	1	2	0
Yuka (4.36)	2	N/A	1	0	1	N/A	0
Zero (5.32.0)	2	4	4	2	1	0	0

N/A = not applicable due to app category.

**Table 5:** Number of features within paid and/or premium version nutrition-related apps (*n* = 35).

App (Version No.)	Dietary	Tracking	Insights	Technical	Education	Social	Artificial Intelligence
Balance (1.7.8)	1	2	2	0	2	1	0
Bariatric Meal Timer (1.2)	N/A	1	N/A	1	0	N/A	0
Blood Type Diet^®^ (2.6.8)	2	0	1	2	2	1	0
Caffiend (3.2.2)	3	2	3	2	0	0	0
Carb Manager (7.5.5)	6	5	7	2	2	2	1
Centr (6.7.2)	1	2	4	1	1	1	0
Cronometer (4.19.5)	6	4	7	2	0	2	0
Empty Fasting (1.1.1)	0	1	1	1	0	0	0
Fast Tract Diet (2.7)	3	1	4	2	1	0	0
Fastic (1.165.0)	6	4	6	2	2	1	3
FatSecret (9.32)	6	4	6	2	2	1	1
Fitbit (4.13)	4	4	4	3	2	2	0
Fitness Buddy+ (5.410)	2	3	5	2	2	0	0
Food Additives Checker (5.1.0)	N/A	N/A	N/A	0	1	N/A	0
Foodvisor (5.15.0-1)	5	3	7	1	2	0	1
Gluten Free Ingredient List (4.0)	N/A	N/A	1	0	1	N/A	0
HealthifyMe (11.1.0)	4	4	7	1	2	0	2
HitMeal (1.34)	6	4	4	1	2	0	1
kJ 2 Cal (1.0.1)	N/A	N/A	N/A	0	1	N/A	0
Lifesum (18.3.0)	6	5	7	1	1	0	0
Lose It! (16.2.000)	7	5	6	2	1	2	1
MacroFactor (2.6.8)	6	3	6	2	1	1	2
My Macros+ (2024.04)	6	1	5	2	0	1	1
MyFitnessPal (24.10.0)	7	5	6	2	2	2	1
MyNetDiary (9.11)	7	4	7	2	2	2	1
mySymptoms (5.60)	3	3	3	0	0	0	1
Noom (12.9.0)	5	4	5	2	2	1	0
Phatt (1.3.39)	1	2	3	2	2	1	0
Pocket Cal/kJ Pro (2.2)	N/A	2	3	0	1	0	0
Potassium Counter & Tracker (2.10.6)	5	2	3	2	1	0	1
Tap & Track (8.2)	3	3	6	1	0	0	0
Virtual Gastric Band Hypnosis (3.2.4)	1	2	3	1	1	1	0
Weight Diary (13.0)	N/A	1	3	2	0	0	0
YAZIO (10.6.1)	5	5	7	2	2	2	1
Zero (5.32.0)	2	4	5	2	1	0	0

N/A = not applicable due to app category. Note that apps that were free to download but required payment to use them are listed in this table.

**Table 6:** Macronutrients output (as % energy) from the three-day food records and nutrition-related apps (*n* = 15) for three diet types—Western, Asian, and Recommended.

	Western Diet					Asian Diet					Recommended Diet				
Source (Version No.)	Protein (% E)	Total Fat (% E)	Sat Fat (% E)	CHO (% E)	Sugar ^1^ (% E)	Protein (% E)	Total Fat (% E)	Sat Fat (% E)	CHO (% E)	Sugar (% E)	Protein (% E)	Total Fat (% E)	Sat Fat (% E)	CHO (% E)	Sugar (% E)
*3-day food records*	*17*	*31*	*12*	*45*	*20*	*19*	*34*	*10*	*46*	*12*	*20*	*27*	*7*	*48*	*20*
Carb Manager (7.5.5)	18	35	N/A	45	N/A	16	31	N/A	46	N/A	20	23	N/A	63	N/A
Cronometer (4.19.5)	15	31	12	52	20	15	27	8	51	20	21	24	6	58	16
Easy Diet Diary (6.0.28)	17	32	14	44	21	15	30	9	54	13	21	26	7	48	21
Fastic (1.165.0)	14	31	13	53	41	11	32	11	69	9	21	28	14	59	15
FatSecret (9.32)	17	36	12	44	15	15	26	8	60	15	20	27	8	55	19
Fitbit (4.13)	16	35	13	44	17	19	29	9	51	14	18	27	7	57	15
Foodvisor (5.15.0-1)	15	34	13	46	12	15	27	8	50	13	22	23	N/A	52	N/A
HealthifyMe (11.1.0)	15	33	N/A	47	N/A	14	32	N/A	52	N/A	21	24	N/A	55	N/A
HitMeal (1.34)	16	36	N/A	44	N/A	18	27	N/A	62	N/A	20	27	N/A	53	N/A
Lifesum (18.3.0)	15	37	N/A	41	N/A	19	24	N/A	57	N/A	20	21	N/A	60	N/A
Lose It! (16.2.000)	14	31	12	46	22	11	15	5	29	7	21	30	8	54	10
MacroFactor (2.6.8)	16	36	14	45	18	14	31	N/A	55	N/A	20	22	5	60	17
MyFitnessPal (24.10.0)	19	33	12	43	14	17	31	7	56	8	21	24	5	54	19
MyNetDiary (9.11)	16	37	13	45	N/A	17	23	8	53	N/A	20	23	6	58	N/A
YAZIO (10.6.1)	15	34	N/A	44	N/A	15	28	N/A	52	N/A	19	27	N/A	57	N/A
Average of apps	16	34	13	45	20	16	28	8	53	12	20	25	7	56	17

Note: E = energy; Sat = saturated; CHO = carbohydrate; N/A = Not applicable. ^1^ Sugar reported is total sugar as % energy.

**Table 7:** Micronutrients (dietary fibre, calcium, and iron density) from the three-day food records and nutrition-related apps (*n* = 12) for all three diet types—Western, Asian, and Recommended.

	Western Diet				Asian Diet				Recommended Diet			
Source (Version No.)	Fibre Density	Calcium Density	Iron Density	Sodium Density	Fibre Density	Calcium Density	Iron Density	Sodium Density	Fibre Density	Calcium Density	Iron Density	Sodium Density
*3-day food records*	*2*	*96*	*1*	*278*	*2*	*75*	*1*	*402*	*5*	*154*	*1*	*201*
Carb Manager (7.5.5)	2	N/A	N/A	N/A	2	N/A	N/A	N/A	5	N/A	N/A	N/A
Cronometer (4.19.5)	2	75	1	876	2	75	1	875	5	140	1	159
Easy Diet Diary (6.0.28)	2	87	1	279	3	45	1	461	6	173	2	174
Fastic (1.165.0)	2	21	0	9511	1	17	0	45,524	5	36	0	107,346
FatSecret (9.32)	1	N/A	N/A	263	2	N/A	N/A	327	4	N/A	N/A	307
Fitbit (4.13)	1	67	21	318	2	41	22	549	4	71	29	464
Foodvisor (5.15.0-1)	2	29	0	231	2	50	0	525	4	76	2	200
HealthifyMe (11.1.0)	2	N/A	N/A	N/A	2	N/A	N/A	N/A	5	N/A	N/A	N/A
Lose It! (16.2.000)	2	N/A	0	227	1	N/A	N/A	224	4	6	0	211
MacroFactor (2.6.8)	2	66	1	355	N/A	N/A	N/A	N/A	5	103	2	231
MyFitnessPal (24.10.0)	1	8	3	243	2	126	1	416	4	258	1	153
MyNetDiary (9.11)	2	103	N/A	273	2	24	N/A	315	5	120	0	196

Note. All values are reported as g/MJ of energy for dietary fibre or mg/MJ of energy for calcium, iron and sodium. The Asian and Recommended diets did not include any alcoholic drinks, and so alcohol has not been reported.

**Table 8:** Selected apps (*n* = 7) that included an AI-enabled food image recognition feature for food logging. Accuracy was calculated as the number of food components correctly identified divided by the total number of food components (*n* = 39) for each app.

App (Version No.)	Does It Automatically Estimate Calories from the Recognised Food/Drink? (Y/N)	Food Components Correctly Identified (*n* = 39)	Accuracy (%)
Lose It! (16.2.000)	N	18	46
Fatsecret (1.165.0)	N	18	46
Hitmeal (1.34)	N	24	62
Foodvisor (5.15.0-1)	Y	34	87
HealthifyMe (11.1.0)	Y	35	90
Fastic (1.165.0)	Y	36	92
MyFitnessPal (24.10.0)	Y	38	97

**Table 9:** Energy output from AI-enabled food image recognition in apps (*n* = 4) compared to the food records for single component food items.

Food Components	Food Record Reference (kJ)	MyFitnessPal, Energy Difference (kJ)	MyFitnessPal % Energy Difference	Foodvisor, Energy Difference (kJ)	Foodvisor % Energy Difference	HealthifyMe, Energy Difference (kJ)	HealthifyMe % Energy Difference	Fastic, Energy Difference (kJ)	Fastic % Energy Difference
Instant Cup Noodles-Nongshim Shin	1195	N/A	N/A	N/A	N/A	207	17	18	2
Boiled Egg	235	50	21	12	5	87	37	50	21
Latte Coffee with Full Cream Milk	430	−7	−2	−242	−56	319	74	198	46
Pepsi Max Can	6	N/A	N/A	N/A	N/A	N/A	N/A	−6	−100
Potato Chips	487	136	28	−77	−16	−56	−12	659	135
Green Tea	14	−14	−100	−14	−100	N/A	N/A	−14	−100
Iced Tea (Black Tea)	121	−111	−92	−121	−100	N/A	N/A	297	246
Savoury Biscuit (Sakata Seaweed cracker)	334	N/A	N/A	N/A	N/A	N/A	N/A	294	88
Galbi-Korean BBQ Marinated Beef Short Ribs	1820	−1109	−61	−1176	−65	N/A	N/A	N/A	N/A
Kimchi	29	65	224	N/A	N/A	−12	−42	34	116
***Mean energy difference against reference (SD)***		*−254 (480)*	*−3 (97)*	*−229 (395)*	*−47 (41)*	*−20 (316)*	*8 (40)*	*111 (265)*	*44 (102)*

N/A = not identified, not applicable.

**Table 10:** Energy output from AI-enabled food image recognition in apps (*n* = 4) compared to the food records for mixed food component dishes.

Name of Dish, Component	Food Record Reference (kJ)	MyFitnessPal, E Difference (kJ)	MyFitnessPal E Difference (%)	Number of Components Identified	Foodvisor, E Difference (kJ)	Foodvisor E Difference (%)	Number of Components Identified	HealthifyMe, E Difference (kJ)	HealthifyMe E Difference (%)	Number of Components Identified	Fastic, E Difference (kJ)	Fastic E Difference (%)	Number of Components Identified
**Western diet mixed dishes**													
**Eggs on toast with butter**	1623	−569	−35%		−556	−34%		155	10%		−368	−23%	
White Toast	736	−108	−15%	2/3, omitted butter	−71	−10%	3/3 identified	76	10%	3/3 identified	N/A	N/A	Didn’t identify individual components
Fried Egg (fried in oil)	306	120	39%		−59	−19%		230	75%		N/A	N/A	
Butter, Regular fat	581	N/A	N/A		−426	−73%		−150	−26%		N/A	N/A	
**Spaghetti Bolognese**	1489	−192	−13%		−443	−30%		369	25%		394	26%	
Pasta Sauce	111	182	164%	3/3 identified	N/A	N/A	Didn’t identify individual components	N/A	N/A	Didn’t identify individual components	N/A	N/A	Didn’t identify individual components
Spaghetti pasta	803	494	62%		−510	−64%		N/A	N/A		N/A	N/A	
Beef (minced)	690	21	3%		N/A	N/A		N/A	N/A		N/A	N/A	
**Hamburger**	2299	−1044	−45%		15	1%		−667	−29%		−416	−18%	
Bun	836	N/A	N/A	Didn’t identify individual components	N/A	N/A	Didn’t identify individual components	N/A	N/A	Didn’t identify individual components	N/A	N/A	Didn’t identify individual components
Beef Patty	300	N/A	N/A		N/A	N/A		N/A	N/A		N/A	N/A	
Cheese	357	N/A	N/A		N/A	N/A		N/A	N/A		N/A	N/A	
Lettuce	9	N/A	N/A		N/A	N/A		N/A	N/A		N/A	N/A	
Tomato	22	N/A	N/A		N/A	N/A		N/A	N/A		N/A	N/A	
**Asian diet mixed dishes**													
**Beef and Vegetable Stir Fry**	1503	−591	−39%		−708	−47%		999	66%		−185	−12%	
Beef	519	192	37%	4/4 identified	418	81%	Identified 3/4 components, identified bok choy as green asparagus or zucchini	N/A	N/A	Didn’t identify individual components	N/A	N/A	Didn’t identify individual components
Bok Choy	308	−300	−97%		N/A	N/A		N/A	N/A		N/A	N/A	
Carrots	75	30	41%		101	135%		N/A	N/A		N/A	N/A	
Mushrooms	67	21	31%		180	267%		N/A	N/A		N/A	N/A	
**Beef Pho**	1424	233	16%		701	49%		170	12%		40	3%	
Rice noodles	829	N/A	N/A	Didn’t identify individual components	N/A	N/A	Didn’t identify individual components	N/A	N/A	Didn’t identify individual components	N/A	N/A	Didn’t identify individual components
Beef	330	N/A	N/A		N/A	N/A		N/A	N/A		N/A	N/A	
Bean sprouts	6	N/A	N/A		N/A	N/A		N/A	N/A		N/A	N/A	
**Bibimbap**	1100	2967	270%		92	8%		1373	125%		−54	−5%	
Brown Rice (steamed)	798	N/A	N/A	Didn’t identify individual components	173	22%	3/6 components identified, didn’t identify spinach and bean sprout correctly, omits gochujang paste	N/A	N/A	Didn’t identify individual components	N/A	N/A	Identified Bibimbap as seasoned spinach salad, carrot, bean sprout and brown rice, 4/6 components identified but didn’t have energy for each individual component
Zucchini	29	N/A	N/A		88	304%		N/A	N/A		N/A	N/A	
Bean sprouts	21	N/A	N/A		N/A	N/A		N/A	N/A		N/A	N/A	
Spinach	16	N/A	N/A		N/A	N/A		N/A	N/A		N/A	N/A	
Grated carrot	102	N/A	N/A		3	3%		N/A	N/A		N/A	N/A	
Gochujang paste	3	N/A	N/A		N/A	N/A		N/A	N/A		N/A	N/A	
**Pearl Milk Tea (Full sugar)**	2594	−1430	−55%		N/A	N/A		−1966	−76%		−1264	−49%	
Milk Tea	N/A	N/A	N/A	Didn’t identify individual components	N/A	N/A	Identified bubble tea as cappuccino or plain yoghurt	N/A	N/A	Didn’t identify individual components	N/A	N/A	Didn’t identify individual components
Pearls	N/A	N/A	N/A		N/A	N/A		N/A	N/A		N/A	N/A	

Note: E = energy, N/A not applicable, the recipe or food component was not correctly identified by the app.

## Data Availability

The original contributions presented in the study are included in the article/Supplementary Material, further inquiries can be directed to the corresponding author.

## Supplementary Materials

The following supporting information can be downloaded at: https://www.mdpi.com/article/10.3390/nu16152573/s1, Table S1: Sample three-day food records based on three dietary patterns (Western, Asian, and Recommended diet based on the Australian Dietary Guideline recommendations); Table S2: Dishes and drinks derived from Western and Asian diet Day 1 for AI-enabled food image recognition analysis, with their respective amounts; Figure S1: Food and drink images were captured using a smartphone and uploaded into AI-enabled food image recognition apps (Total of 22 images, 16 food images, and 6 drink images): (A) Single-component food and drink images (*n* = 10 food images and *n* = 5 drink images); and (B) Mixed dishes (*n* = 6 images of mixed food dishes and *n* = 1 image of mixed drinks).
